# Sleep Disorders and Cognitive Function in Multiple Sclerosis: A Systematic Review of Polysomnographic Studies and Implications for Neurorehabilitation Strategies

**DOI:** 10.3390/life16040699

**Published:** 2026-04-21

**Authors:** Laura-Elena Cucu, Laura-Cristina Baciu, Cristina Grosu, Emilian Bogdan Ignat, Carmen Marinela Cumpăt, Mihai Roca, Costin Chirica, Gabriela Popescu, Maria-Magdalena Leon

**Affiliations:** 1 Doctoral School, “Grigore T. Popa” University of Medicine and Pharmacy, 700115 Iasi, Romania; dudau.laura-elena@d.umfiasi.ro (L.-E.C.);; 2 Clinical Rehabilitation Hospital, 700661 Iasi, Romania; 3 Department of Neurology, “Grigore T. Popa” University of Medicine and Pharmacy, 700115 Iasi, Romania; 4 Department of Medical Specialties III, “Grigore T. Popa” University of Medicine and Pharmacy, 700115 Iasi, Romania; 5 Department of Medical Specialties I, “Grigore T. Popa” University of Medicine and Pharmacy, 700115 Iasi, Romania

**Keywords:** sleep, neurorehabilitation, multiple sclerosis, cognition, polysomnography

## Abstract

Cognitive rehabilitation represents a cornerstone of disease management in multiple sclerosis (MS), targeting the progressive cognitive decline that affects a significant proportion of patients. Despite growing evidence supporting its clinical utility, rehabilitation outcomes remain variable, and identifying modifiable factors that limit its efficacy has become a research priority. Sleep disorders are common in MS and have been increasingly linked to cognitive impairment, yet evidence based on objective polysomnographic assessment remains limited, and the specific parameters that influence cognitive function are poorly understood. This review synthesizes evidence from polysomnographic studies examining how sleep disturbances influence cognitive performance in MS patients. Following a systematic search of PubMed, EMBASE, and the Cochrane Library, 488 patients were included. Sleep fragmentation, reduced sleep efficiency, and oxygen desaturation indices were associated with impairments in attention, information processing speed, and verbal memory, with nocturnal hypoxia emerging as a potentially important mechanism of cognitive impairment. These findings suggest that identifying and treating sleep disorders may be essential for optimizing cognitive rehabilitation outcomes in MS. Further longitudinal studies are needed to determine whether addressing sleep pathology can enhance rehabilitation efficacy and preserve cognitive function over time.

## 1. Introduction

Multiple sclerosis (MS) is a demyelinating disease characterized by both inflammation and neurodegeneration, representing a major cause of disability in young adults. Rehabilitation in MS has traditionally focused on physical impairments, including gait and balance disorders, fatigue, and spasticity [1,2]. These interventions aim to reduce disability, promote neuroplasticity, and improve quality of life through multidisciplinary approaches combining physiotherapy, occupational therapy, and cognitive rehabilitation [2]. However, cognitive dysfunction represents an equally disabling yet comparatively underaddressed dimension of the disease, affecting 32.5–70% of patients [3,4,5,6] even in the early stages [7] and worsening with disease progression [8]. This impairment significantly impacts daily functioning, resulting in lower quality of life [9] and higher unemployment rates [10]. Given the current lack of treatment for cognitive dysfunction, there is growing interest in identifying modifiable factors that could influence cognitive performance in these patients.

Sleep disturbances, though frequently overlooked, occur more often in PwMS (people with multiple sclerosis) compared to the general population, with approximately 50% of PwMS reporting such issues [11]. Polysomnographic studies have also revealed objective sleep abnormalities: A 2024 meta-analysis reported significant decreases in sleep efficiency and stage N2 sleep percentage, alongside increases in wake after sleep onset, periodic limb movement index, and periodic limb movement arousal index compared to healthy controls [12]. Both symptomatic medications and disease-modifying therapies may contribute to these disturbances; interferons, for instance, were associated with reduced sleep efficiency particularly when administered in the evening, possibly due to interferon-induced flu-like symptoms or alterations in interleukin levels [13,14]. Numerous comorbidities common in PwMS, such as nocturia, paresthesia, pain, depression, anxiety, and nighttime leg cramps, further compound sleep pathology [15,16,17]. Sleep disorders, being a potentially modifiable factor, might therefore contribute to cognitive dysfunction in PwMS, and identifying and addressing them could influence long-term prognosis.

To date, the number of studies examining this relationship remains limited, and most rely on self-reported sleep measures. Two recent systematic reviews combining subjective and objective sleep assessment have consistently identified significant associations between objectively measured sleep parameters and cognitive impairment. Golabi et al. 2024 [4] reviewed 35 studies and found significant associations with objective sleep assessment but limited evidence for self-reported sleep quality. Similarly, Hughes et al. 2018 [11] reviewed 12 studies and reported that objective sleep measures predicted cognitive impairments in information processing speed and attention, whereas self-reported sleep disturbance predicted only subjective cognitive complaints. Neither review focused exclusively on polysomnographic data. Given that both reviews identified associations primarily with objective sleep measures, and considering the documented subjective–objective discrepancies in sleep assessment [11,18,19,20], the present review focused exclusively on polysomnographic data, the gold standard for sleep assessment. The objective was to provide a detailed analysis of sleep architecture parameters and sleep-disordered breathing indices and their relationship with specific cognitive domains, with implications for cognitive rehabilitation strategies in MS.

## 2. Materials and Methods

This systematic review was conducted and reported in accordance with the Preferred Reporting Items for Systematic Reviews and Meta-Analyses (PRISMA) 2020 guidelines [21]. The completed PRISMA checklist is provided in Supplementary Table S1. The review protocol was retrospectively registered on the Open Science Framework (https://doi.org/10.17605/OSF.IO/XGVKQ).

We searched PubMed, EMBASE, and the Cochrane Library from inception to 31 December 2025, using a combination of Medical Subject Headings (MeSH) and free-text keywords. The complete search strings, including Boolean operators and field restrictions, are detailed in Supplementary File S1. Only peer-reviewed publications in English were included. Reference lists of all eligible studies were manually screened for additional records. Gray literature was not searched.

The research question was structured according to the following PICO framework.

P (Population): Adults with confirmed MS diagnosis.

I (Intervention/Exposure): Objective sleep assessment via polysomnography.

C (Comparator): Not applicable.

O (Outcome): Cognitive function assessed by validated neuropsychological instruments.

Inclusion criteria required that all enrolled patients were adults with a confirmed diagnosis of multiple sclerosis according to the McDonald Criteria [22]. Studies that did not specify diagnostic standards but relied on physician-determined MS status through clinical evaluation and neuroimaging were also included. Objective sleep measurements obtained through polysomnography were mandatory alongside cognitive assessment performed using validated neuropsychological instruments administered by trained examiners. Only studies reporting associations between polysomnographic sleep parameters and cognitive function in PwMS were included.

Studies were excluded if they were review articles, abstracts, case reports, case series, editorials, opinion statements, comments or guidelines, or publications not in English. Studies reporting only subjective cognitive function or patient-perceived cognitive deficits without objective neuropsychological testing were also excluded. A detailed list of studies excluded at full-text screening is provided in Supplementary Table S2.

Two reviewers (L.-E.C. and L.C.B.) independently screened all titles and abstracts retrieved from the database searches, followed by full-text assessment of potentially eligible studies, data extraction, and methodological quality assessment. Disagreements at any stage were resolved through discussion and, when consensus could not be reached, by a third reviewer (M.M.L.).

The following data were extracted from each included study: demographic and clinical characteristics of participants (age, sex, MS subtype, EDSS score), polysomnographic parameters (sleep efficiency, sleep onset latency, wake after sleep onset, sleep stage percentages, AHI, ODI, and minimum oxygen saturation), inclusion and exclusion criteria, OSA prevalence, cognitive assessment instruments used, cognitive domains evaluated, and sleep–cognition associations.

Given that all the data extracted from included studies were cross-sectional, methodological quality was assessed using the Newcastle–Ottawa Scale for cross-sectional studies (NOS-xs) [23], with results presented in Supplementary Table S3.

A meta-analysis was not performed due to significant heterogeneity in the definitions of exposure and outcome variables across included studies. Specifically, studies reported different polysomnographic parameters regarding sleep-disordered breathing indices in varying combinations. Moreover, different cognitive assessment instruments were used for the same cognitive domains, precluding meaningful data pooling.

## 3. Results

**Figure 1:**
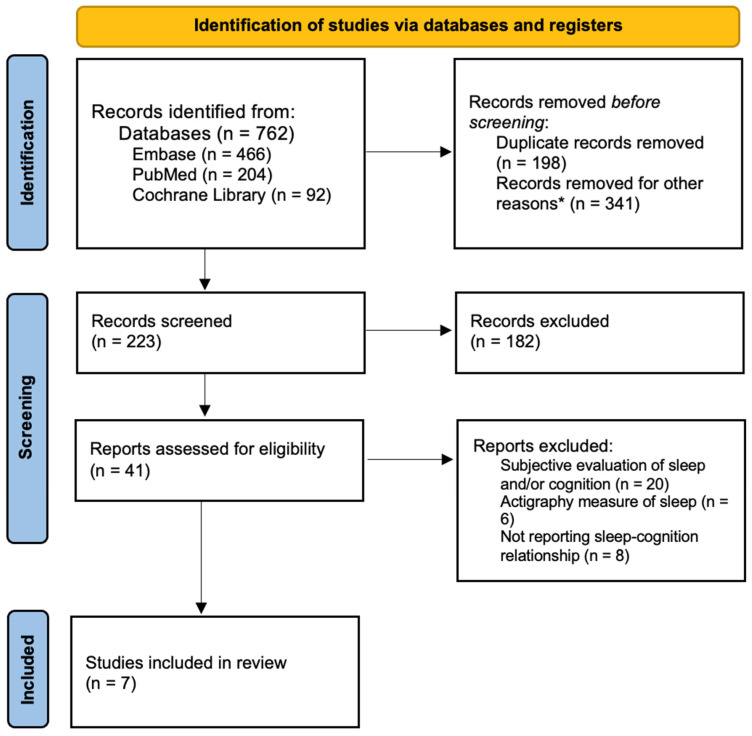
Flow diagram of study selection process. * Animal studies (n = 10), non-English studies (n = 8), conference papers (n = 220), reviews (n = 53), correction (n = 1), editorials (n = 1), commentaries (n = 1), study protocols (n = 37), book chapter (n = 1), erratum (n = 9).

The database search retrieved 762 records. After removal of duplicates and exclusions prior to screening, 223 records were assessed by title and abstract, and 41 proceeded to full-text assessment. Of these, 34 were excluded due to use of only subjective sleep evaluation (n = 20), use of actigraphy as the sole sleep measure (n = 6), or failure to report a sleep–cognition relationship (n = 8). Seven studies met the inclusion criteria and were incorporated into the final review (Figure 1).

A comprehensive overview of the methodological characteristics of all 7 included studies is provided in Table 1.

The methodological quality of included studies was moderate to high, with NOS-xs scores ranging from 5 to 8 out of 9. Four studies demonstrated low risk of bias, scoring 7 or 8 out of 9, while three studies [24,25,28] received a score of 5 or 6 out of 9, indicating moderate risk of bias (Supplementary Table S3).

### 3.1. Demographic Characteristics

The reviewed studies, published between 2015 and 2024, were all cross-sectional in design, with the exception of McNicholas et al. 2021 [26], which employed a longitudinal design. However, only baseline cross-sectional data were extracted for the purposes of this review. The cumulative sample size encompassing all studies amounted to 488 participants (range 23–131). Within this cohort, the majority of patients were female (55–75%). Racial and ethnic demographic data were largely absent from the studies. Most studies concentrated on patients with relapsing-remitting multiple sclerosis (55.75–100%), while one study evaluated predominantly patients with progressive forms [28]. The prevalence of obstructive sleep apnea (OSA) reported in all studies except one [27] showed considerable heterogeneity, ranging from 15.04 to 86.3%.

### 3.2. Patient Selection

The inclusion and exclusion criteria across studies were diverse. Four articles restricted enrollment to patients with lower disability levels (EDSS < 6.5) [24,25,26,27], whereas one study included more severely disabled patients (EDSS ≥ 6.5) [28]. Some studies applied symptom-specific recruitment strategies. One study [5] used a patient-initiated approach, recruiting individuals who spontaneously asked about sleep or cognitive problems during routine clinical encounters. Another study [26] excluded patients treated with fatigue-modifying medications (modafinil or amantadine). One study [24] specifically selected symptomatic patients with elevated scores on fatigue measures (Fatigue Severity Scale—FSS, Modified Fatigue Impact Scale—MFIS) or sleepiness assessment (Epworth Sleepiness Scale—ESS). Another study [6] focused on obstructive sleep apnea (OSA), enrolling patients with confirmed diagnosis or high risk based on STOP-Bang screening questionnaire, a validated tool for OSA risk assessment.

The authors excluded patients with relapse or corticosteroid therapy in the previous month [6,25], 6 weeks [5], or 3 months [28], those with psychiatric disease [6,25], alcohol or drug abuse [5,25,27], severe cognitive impairment [5,6,24,26,27], and depression assessed using BDI [25,26] or PHQ-9 [5]. Further exclusion criteria were coexisting medical condition [24], such as cardiopulmonary disease [5,6] and specifically severe COPD (chronic obstructive pulmonary disease) [28] or other neurological disorder that may increase the OSA risk [5,6,26,27], sleep apnea treatment [5,6,26], pregnancy [6,28]. Change in DMT was an exclusion criterion in only two studies [6,27].

### 3.3. Sleep Assessment

All patients underwent overnight polysomnography as an objective measure of sleep, with all PSGs scored in agreement with the American Academy of Sleep Medicine (AASM) 2007 criteria [29] in four studies [5,24,25,27] and AASM scoring manual version 2.2 [30] in three studies [6,26,28]. The PSG assessed sleep architecture parameters: Total Sleep Time (TST), Sleep Efficiency (SE), Sleep Onset Latency (SOL), time in each sleep stage (N1, N2, N3, REM), Wake After Sleep Onset (WASO), Total Arousal Index (TAI), and the number of awakenings per hour. Sleep apnea parameters that were measured included: Apnea–Hypopnea Index (AHI), Hypopnea Index (HI), Respiratory Disturbance Index (RDI), Respiratory Event Index (REI), Oxygen Desaturation Index (ODI), and mean and lowest oxygen saturation (MinO_2_). Movement parameters assessed were: Periodic Limb Movement Index (PLMI) and Periodic Limb Movement Arousal Index (PLMAI). Several studies also used subjective measures of sleep such as the Epworth Sleepiness Scale (ESS, n = 6) [6,24,25,26,27,28] and the Pittsburgh Sleep Quality Index (PSQI, n = 2) [27,28].

### 3.4. Cognitive Assessment

**Figure 2:**
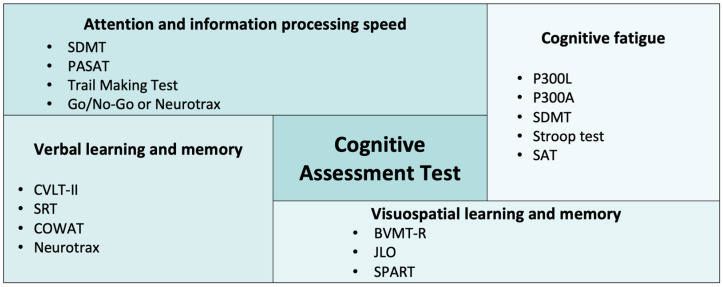
Cognitive assessment tests used in the included studies. Abbreviations: BVMT—Brief Visuospatial Memory Test; COWAT—Controlled Oral Word Association Test; CVLT—California Verbal Learning Test; JLO—Judgment of Line Orientation Test; PASAT—Paced Auditory Serial Addition Test; SAT—Serial Addition Test; SDMT—Symbol Digit Modalities Test; SPART—Spatial Recall Test; SRT—Selective Reminding Test.

Cognitive function was assessed using validated neuropsychological tests across multiple domains (Figure 2).

Global cognition was evaluated using comprehensive batteries including NeuroTrax [24] and the Brief Repeatable Battery of Neuropsychological Tests [27].

Attention and information processing speed were assessed in all seven studies, most commonly using the Symbol Digit Modalities Test (n = 6) [5,6,25,26,27,28] and Paced Auditory Serial Addition Test (n = 3) [5,26,27]. Additional tests included the Trail Making Test [6], Go/No-Go Test [6], and NeuroTrax computerized battery [24].

Verbal learning and memory were evaluated in six studies using the California Verbal Learning Test-II (n = 4) [5,6,26,28], Selective Reminding Test [27], Controlled Oral Word Association Test [6], and NeuroTrax [24].

Visuospatial learning and memory were assessed in five studies using the Brief Visuospatial Memory Test-Revised [5,6,26,28], Judgment of Line Orientation [5,6], and 10/36 Spatial Recall Test [27].

Cognitive fatigue was investigated in one study [25] using performance-based measures (Stroop Test, Symbol Digit Modalities Test, and Sustained Attention Test) with fatigue scores calculated by dividing the 60 s score by the 180 s score. Electrophysiological measures included P300 peak latency and peak amplitude, calculated as a ratio between responses to 150 versus 50 stimuli.

The interval between cognitive assessment and PSG was reported in three studies: 2 weeks [25], 4–8 weeks [24], 48 days [5], while the remaining four studies did not specify this timing [6,26,27,28], with one study [6] allowing an interval of up to 12 months.

### 3.5. Confounding Factors

Most of the included studies adjusted the results for age [5,6,24,26,27] and education [5,24,26]. Two studies adjusted for depression symptoms [5,6] or disease duration [5,27], and each one for DMTs [27], MS subtype, sleepiness and AHI [6].

The DMT received at assessment was specified in one study [5], while another adjusted for DMT without reporting which drugs were used [27]. Only one study documented symptomatic medications at baseline [5].

### 3.6. Associations Between Sleep Architecture and Cognition

Global cognition scores on NeuroTrax [24] and Brief Repeatable Battery of Neuropsychological Tests [27] were positively associated with SE in two studies [24,27] and negatively correlated with WASO [27].

Attention and information processing speed demonstrated positive associations with SE [24,27], %REM [27], and TST [5], while SOL [24] and WASO [26] were negatively correlated with test performance.

Verbal learning and memory performance showed variable associations. California Verbal Learning Test-II scores correlated with TAI and TST [5], as well as with %N2, %N3, and SOL [6]. NeuroTrax verbal memory scores demonstrated a negative association with WASO [24].

Visuospatial learning and memory showed limited associations with sleep architecture parameters. Only one study [6] found a correlation between Brief Visuospatial Memory Test-Revised performance and %N2 sleep.

Cognitive fatigue measures demonstrated statistically significant associations with multiple sleep parameters. Performance-based fatigue scores (Stroop Test, Symbol Digit Modalities Test, Sustained Attention Test) correlated with SE, SOL, %N1, %N2, %N3, and WASO. Electrophysiological measures (P300 peak latency and peak amplitude) were associated with SOL, %N1, and %N2 [25].

### 3.7. Associations Between Sleep-Disordered Breathing and Cognition

Four of seven studies found no correlation between sleep apnea measures and cognition [24,25,27,28], but one study reported REI as the only indicator for assessing sleep apnea severity [25]. There was a statistically significant correlation between MinO_2_ [5], ODI [5,6], OAI [6], CAI [6], RDI [5] and attention, and executive functioning, tested with Symbol Digit Modalities Test [5,6], Paced Auditory Serial Addition Test [5] and Trail Making Test [6]. OAI [6], RDI [5], and MinO_2_ [26] were correlated with verbal memory and executive function, evaluated using California Verbal Learning Test-II. One study [5] found an association between ODI and MinO_2_ and visual memory, assessed with Brief Visuospatial Memory Test-Revised.

## 4. Discussion

Compared to other neurological conditions, MS does not follow a traditional rehabilitation trajectory due to the variability and unpredictability of its clinical course, and the optimal timing for rehabilitative interventions remains poorly defined. Current management emphasizes patient education regarding disease-related deficits, the adoption of compensatory strategies, and environmental adaptation [31]. Cognitive rehabilitation has emerged as an area of growing interest, with evidence supporting a stepwise approach, addressing foundational skills such as attention and orientation before progressing to higher-order functions including memory [32].

Sleep assessment can be done through questionnaires, a subjective measure, or objectively through polysomnography or actigraphy. Questionnaires are the most commonly used method, being low-cost, comfortable for patients, and requiring no specialized equipment.

Cognitive impairment in PwMS is multifactorial, with established risk factors including education level, late-onset disease, primary progressive disease course, comorbid neuropsychiatric conditions, and genetic predisposition [8,33,34,35,36]. Beyond these, cognitive performance in PwMS is further influenced by depression, fatigue, and disability level [37], as well as disease duration and medication use [38], all of which represent potential confounders in sleep–cognition research. However, these variables were inconsistently controlled for across included studies, and no study adjusted for symptomatic medication use, such that residual confounding cannot be excluded and the observed sleep–cognition associations should be interpreted accordingly.

Furthermore, the results should be interpreted cautiously given differences in PSG scoring criteria and the considerable variability in inclusion criteria across studies. Most studies enrolled patients with lower disability levels [24,25,26,27], while one specifically targeted more severely disabled patients [28]. Additionally, some studies applied symptom-specific recruitment strategies, selectively enrolling patients with significant fatigue [24], sleep or cognitive complaints [5], or confirmed OSA diagnosis or high OSA risk [6], which contributed to the wide variation in OSA prevalence across studies, ranging from 15.04% to 86.3%. The variable time interval between polysomnographic recording and cognitive assessment, ranging from 2 weeks to 8 weeks in the three studies that reported it, with four studies not reporting this information, represents an additional source of variability. Among the latter, one study [6] permitted the use of existing PSG data obtained up to 12 months prior to cognitive testing. Given the fluctuating nature of MS symptoms, sleep quality, fatigue, and mood over several weeks, this time lag may have further influenced the observed associations. Taken together, these differences limit the comparability of findings across studies and their generalizability to the broader MS population and should be considered when interpreting the observed associations.

### 4.1. Sleep Architecture and Cognitive Function in MS

The most robust findings across studies indicated associations between sleep efficiency and attention, information processing speed, and global cognition [24,27]. Among all cognitive domains examined, attention and information processing speed, verbal memory and learning, and cognitive fatigue showed the most frequent associations with sleep architecture parameters, suggesting that sleep architecture may influence these cognitive domains in PwMS, though causal relationships cannot be established given the cross-sectional nature of the included studies. Notably, these domains are also known to be independently affected by depression in PwMS [39], which was not consistently controlled for across studies, raising the possibility that depressive symptoms may have confounded some of the observed associations.

Riccitelli et al. 2022 [27], rated as low risk of bias, studied the relationship between sleep, psychiatric symptoms (depression and anxiety) and cognitive impairment. There was a statistically significant association between trait anxiety, WASO and memory, but without any association with depression.

One of the included studies [25] analyzed cognitive fatigue in a cohort of 113 patients, and observed that PwMS with reduced SE, increased SOL and WASO had more cognitive fatigue. When comparing with questionnaire results, they found that patients did not perceive their sleep disturbances or cognitive fatigue and suggested routine PSG evaluation in PwMS. The moderate risk of bias of this study warrants cautious interpretation of these findings.

Sater et al. 2015 [24] explored the relationship between sleep, fatigue, depression and cognition in PwMS during a period when they were not receiving DMT treatment and before starting Natalizumab therapy. This study is notable in documenting the symptomatic medications used by participants at the time of assessment, though assigned a moderate risk of bias. Sleep studies showed abnormalities in nearly all patients (30/32), with polysomnography and multi-sleep latency tests showing marked reduction in N3 stage duration, consistent with previous findings [6,40]. This is relevant as N3 sleep has been implicated in learning and memory consolidation.

### 4.2. Obstructive Sleep Apnea, Hypoxia, and Cognitive Outcomes

Sleep apnea severity indices (AHI, RDI, REI) showed inconsistent associations with cognitive function across studies. However, hypoxia-related parameters (ODI, MinO_2_) demonstrated significant associations with verbal memory, attention, and information processing speed, suggesting that intermittent hypoxia may be a more relevant contributor of cognitive impairment than apnea frequency in PwMS.

Braley et al. 2016 [5] were the first to find an association between OSA and cognition in PwMS with sleep or cognition complaint. In their study, sleep-disordered breathing parameters were correlated with attention and information processing speed, delayed visual memory, verbal memory and executive functions. The authors suggested that apnea in MS patients would impact predominantly inhibition and semantic organization among executive functions. From the two possible mechanisms that explain the link between cognitive dysfunction and OSA, namely sleep fragmentation and hypoxia, they favored the latter as the more likely explanation. Additionally, they found no association between fatigue questionnaire scores or sleepiness scales and cognitive dysfunction, suggesting that cognitive impairment in PwMS with sleep disorders cannot be explained solely through fatigue and somnolence. Sleep fragmentation and hypoxia may contribute to cognitive decline through inflammatory mechanisms, with supporting evidence from non-MS populations [41,42], though causal conclusions cannot be drawn from the available cross-sectional evidence.

McNicholas et al. 2021 [26] investigated the relationship between sleep parameters and cognition in PwMS reporting significant fatigue. There was no correlation between sleep apnea parameters and cognition, but verbal memory correlated with MinO_2_. This was the only study that analyzed the effect of OSA treatment (either sleep position therapy or CPAP) on cognition in PwMS. Despite including only 6 treated patients, they showed better results in all tests, except for visuospatial memory and only in verbal memory was the improvement statistically significant. Adjustment was made for age and education level, yet neuropsychiatric symptoms such as depression, which may fluctuate during the treatment period, were not accounted for, limiting the ability to attribute the observed cognitive improvements solely to hypoxia correction. While this study focused on cognitive outcomes, Mazerolle et al. 2024 [43], a study not included in this review as it did not examine the relationship between sleep and cognition, examined long-term CPAP effects on fatigue in PwMS with OSA. Following a randomized controlled trial, 28 patients were followed for approximately three years. Using validated questionnaires, CPAP-adherent patients demonstrated significant improvements in fatigue severity, motor and cognitive fatigue, sleep quality, depressive symptoms, and quality of life, whereas non-adherent patients showed no improvements. Although this study did not directly assess cognitive function with neuropsychological tests, the improvement in cognitive fatigue suggests potential cognitive benefits of OSA treatment in MS.

Both the impairment of verbal memory in those with sleep-disordered breathing [26] and its significant improvement after treatment could suggest a direct link between them. However, whether such a relationship truly exists remains unclear, as most studies analyzed did not find this association. Even Maillart et al. 2024 [28], which enrolled a population at particularly high risk for OSA, predominantly progressive MS forms, a disease course previously associated with increased OSA risk alongside male sex, age, obesity and brainstem lesions [44,45], found no correlation between OSA and cognition, though its moderate risk of bias should be noted. In this cohort, OSA was associated with higher BMI and longer disease duration rather than with respiratory muscle weakness, as inspiratory muscle strength and spirometry did not differ significantly between PwMS with and without OSA.

MS and OSA affect similar cognitive domains including executive function and memory, though MS impairs information processing speed [46,47], while OSA affects attention [26,48]. To date, there is no uniform method for assessing cognition in MS, nor standardized criteria for defining cognitive impairment in these patients [49].

The polysomnographic findings of this review demonstrate that oxygen desaturation indices and minimum oxygen saturation were consistently associated with attention, verbal and visuospatial memory, while sleep efficiency and sleep fragmentation correlated with information processing speed deficits.

An important source of variability frequently overlooked in the studies included in this review is the pharmacological treatment of PwMS, whether disease-modifying or symptomatic. Among disease-modifying therapies, interferons were associated with reduced sleep efficiency, fatigue, and insomnia, though switching from evening to morning injections may attenuate these effects [50]. Additionally, symptomatic medications commonly used in PwMS, including corticosteroids, antispastic agents, antidepressants, and stimulants for fatigue management, may independently alter sleep architecture through diverse mechanisms, affecting parameters such as REM sleep, sleep latency, and total sleep time [50]. Most of the included studies either did not report or did not adjust for the specific medications used, with only one study fully documenting both DMT and symptomatic medications at baseline [5], and one adjusting for DMT use without specifying the agents involved [27], limiting the interpretability of the sleep–cognition associations reported in this review.

### 4.3. Mechanisms and Neurorehabilitation Perspectives

To contextualize the polysomnographic associations identified in this review and to frame neurorehabilitation strategies, the following discussion draws on evidence from the broader literature, as these were not directly assessed in the included studies.

Cognitive dysfunction in PwMS results from both white matter damage and decreased gray matter volume [51,52], with thalamic atrophy correlated significantly with information processing speed, memory, and attention [44,53,54,55], which were also the most frequently impaired domains in our review. As the thalamus is thought to be involved in normal slow-wave sleep [56], it may be an anatomical link between sleep and cognitive problems in PwMS.

Sleep abnormalities can contribute to depression, pain, and fatigue [57,58], but they can also influence the inflammatory pathways [59], potentially implicated in MS progression as well [60]. Riccitelli et al. 2022 [27] proposed the dysfunction of the hypothalamic–pituitary axis (HPA) secondary to the stress caused by trait anxiety as a hypothetical mechanism between sleep disturbances and anxiety. HPA impairment could affect both sleep and memory. Given these bidirectional relationships between sleep disturbances and psychiatric comorbidities, addressing coping mechanisms, stress management and cognitive behavioral therapy represents an important component of the therapeutic approach, with demonstrated benefits for depression, anxiety and quality of life in PwMS [9,61,62,63,64]. Supporting this, Abbasi et al. 2016 demonstrated that cognitive behavioral rehabilitation significantly improved sleep quality in women with MS, with effects persisting one month after therapy completion [64].

Cognitive training may also improve sleep quality. Haimov and Shatil 2013 demonstrated that an 8-week personalized cognitive training program reduced sleep onset latency below the insomnia threshold and increased slow-wave sleep duration and sleep efficiency in older adults, with no such improvements observed in the control group [65]. A shorter, 4-week computerized home-based program similarly yielded benefits in sleep quality, insomnia symptoms, and daytime functioning, though of lesser magnitude [66]. Although current evidence is derived from general and older adult populations, these findings support extending cognitive training approaches to PwMS, who frequently experience sleep disturbances.

Fatigue affects approximately 60% of PwMS globally [67], representing one of the most prevalent and disabling symptoms. However, this common symptom may mask underlying sleep disorders, as both conditions share overlapping symptoms including daytime sleepiness, reduced energy, and cognitive complaints. Therefore, routine screening for OSA should be considered in PwMS with persistent fatigue, particularly when accompanied by cognitive complaints.

Current evidence suggests that cognitive rehabilitation in MS yields modest benefits. Meta-analytic data indicate improvements primarily in subjective memory complaints and information processing, while objective gains in visual memory, working memory, and activities of daily living remain limited [68]. These outcomes raise the question of whether unaddressed comorbidities, particularly untreated sleep disorders, may constrain the effectiveness of such programs. Cognitive rehabilitation in MS includes restorative approaches using computerized training programs to reinforce cognitive skills, and compensatory strategies such as external reminders, visualization techniques, and structured memory methods [69]. Evidence from neuroimaging studies suggests that successful cognitive rehabilitation does not broadly expand brain activation into new regions, but rather consolidates and reinforces activity within networks already implicated in the trained function, a pattern associated with measurable gains in objective cognitive performance [70]. Transcranial direct current stimulation may augment these outcomes, with a recent randomized controlled trial showing that home-based brain stimulation significantly improved cognitive outcomes in MS patients, particularly those with greater neurological disability [71].

Although evidence remains limited, identifying and addressing sleep disorders in conjunction with cognitive rehabilitation may optimize therapeutic outcomes in PwMS with cognitive impairment. For nocturnal hypoxia and obstructive sleep apnea, CPAP therapy represents a potential therapeutic option, though evidence directly supporting its cognitive benefits in PwMS remains limited to a single small study [26]. PwMS with OSA may be particularly vulnerable to cognitive impairment from hypoxia due to their pre-existing demyelinating lesions, cortical atrophy, and reduced brain reserve [26]. Beyond CPAP therapy, inspiratory muscle training was also proposed as a potential therapeutic strategy in OSA, with a recent meta-analysis of seven randomized controlled trials demonstrating significant improvements in inspiratory muscle strength, sleep quality, daytime sleepiness, and lung function [72]. Whether these benefits extend to PwMS with OSA remains to be investigated.

For sleep fragmentation and reduced sleep efficiency, structured aerobic exercise represents a potentially promising complementary approach, though evidence specifically in PwMS is still emerging. Structured aerobic exercise may improve sleep architecture through circadian rhythm regulation [73], enhance cerebral oxygenation and vascular function [74,75], and promote neuroplastic reorganization of brain networks [76]. Al-Sharman et al. 2019 demonstrated that a six-week moderate-intensity aerobic exercise program significantly improved both objective and subjective sleep measures in PwMS [77]. Siengsukon et al. 2016 showed that aerobic exercise reduced daytime sleepiness independently of cardiovascular fitness changes, suggesting circadian regulation mechanisms [73]. Cross-sectional evidence from 290 MS patients further supports a dose-dependent relationship between moderate-to-vigorous physical activity and sleep quality [78].

**Figure 3:**
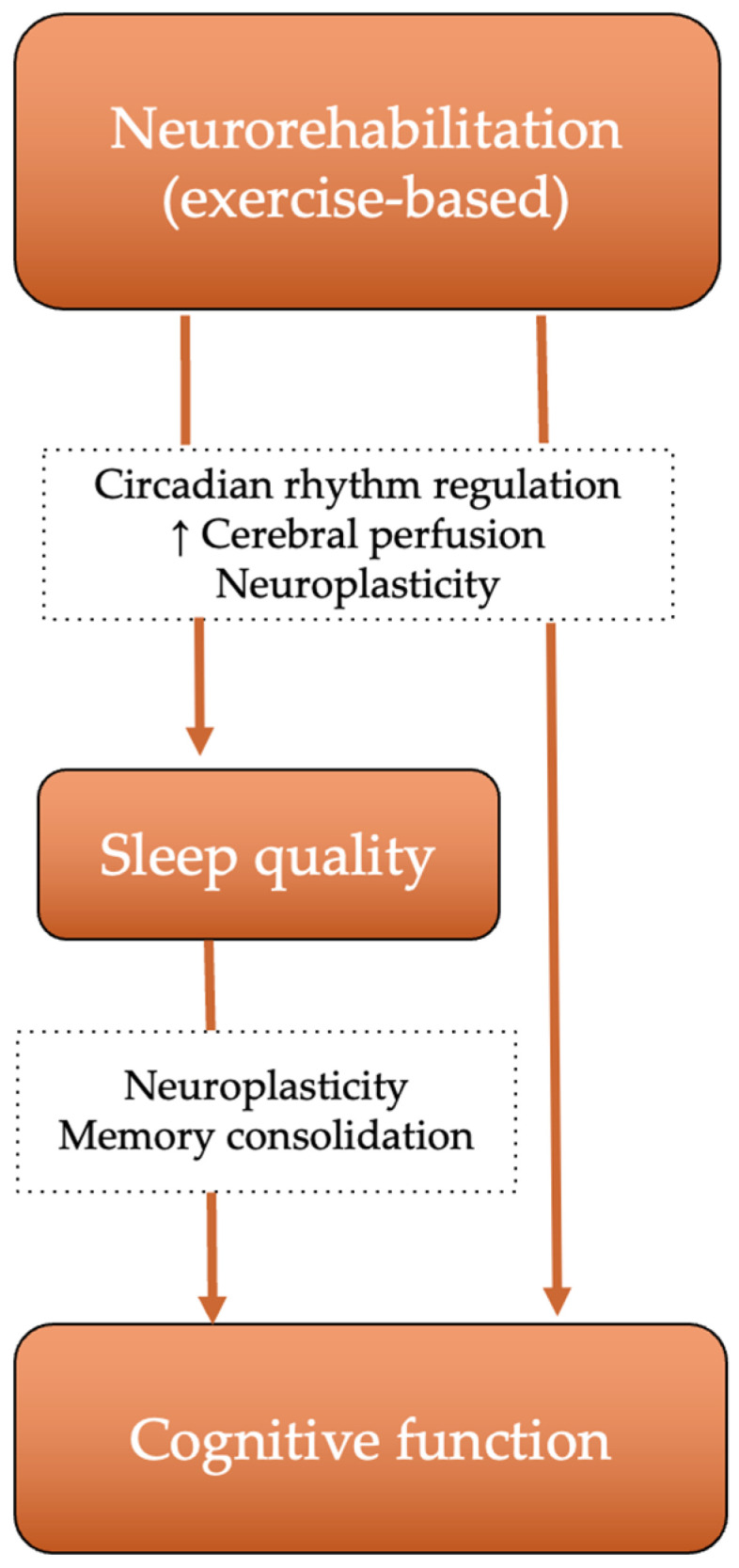
Proposed conceptual framework illustrating the dual pathways by which exercise-based neurorehabilitation enhances cognitive function in PwMS. Exercise-based neurorehabilitation influences cognitive function through both direct mechanisms including neuroplasticity, enhanced cerebral perfusion, and circadian rhythm regulation, and indirect mechanisms mediated by improvements in sleep quality. Improved sleep quality further enhances cognition through memory consolidation and neuroplasticity processes.

Beyond sleep improvement, aerobic exercise may directly enhance cognition through neuroplastic brain network reorganization. Stellmann et al. 2020 demonstrated that 3 months of moderate-intensity exercise enhanced both functional and structural brain connectivity in MS patients, reversing the typical pattern of reduced structural connectivity [76], with potential mechanisms including enhanced cerebral perfusion, increased hippocampal volume, neurogenesis, and reduced inflammation [79]. Furthermore, sleep optimization may actively enhance cognitive rehabilitation outcomes through its role in memory consolidation and neuroplasticity [80]. Based on the mechanistic evidence discussed above, we propose a conceptual framework summarizing these pathways (Figure 3).

These findings support a coherent biological framework positioning sleep and exercise as concurrent modulators of neuroplasticity [73,76,79,80], suggesting that effective cognitive rehabilitation in MS may require integrated protocols combining sleep optimization, structured aerobic exercise, and cognitive training, rather than isolated computerized interventions [68].

### 4.4. Limitations and Future Directions

The primary limitation of this review is the restricted evidence base. Although the focus on polysomnographic studies ensured objectivity, it substantially reduced the number of eligible studies to seven, limiting the statistical strength and generalizability of the conclusions. The included studies were further characterized by small sample sizes, heterogeneous inclusion criteria, lack of longitudinal data, variability in PSG scoring criteria across studies, and inconsistent control for potential confounders including fatigue, depression, and concurrent treatments. The cognitive assessment instruments varied considerably across included studies, differing in cognitive domains assessed, administration protocols, and scoring systems, and may contribute to the inconsistencies observed in reported sleep–cognition associations. The variable time interval between polysomnographic recording and cognitive assessment, ranging from 2 weeks to 8 weeks, with four studies not reporting it, may introduce measurement variability and limit the interpretability of findings across studies. As gray literature was not searched, publication bias cannot be excluded.

Future research should include larger PSG studies and CPAP efficacy trials, with appropriate control for confounding variables, alongside disease subtype comparisons to determine whether relapsing-remitting and progressive forms differ in their susceptibility to sleep-related cognitive dysfunction. Furthermore, randomized controlled trials evaluating the combined effects of exercise-based rehabilitation on both polysomnographic and neuropsychological outcomes in PwMS are warranted. However, before exploring these directions, establishing a standardized cognitive assessment method remains a significant challenge.

## 5. Conclusions

In summary, the available polysomnographic evidence indicates that sleep disturbances in PwMS may be associated with impairments in attention, information processing speed, and verbal memory. Whereas hypoxia-related parameters were more frequently associated with cognitive outcomes than apnea frequency indices, the limited number of studies and methodological heterogeneity preclude firm conclusions. These findings support integrating sleep assessment into the evaluation of cognitively impaired PwMS. Although the evidence base remains insufficient for definitive clinical recommendations, the broader literature suggests that addressing sleep disorders such as nocturnal hypoxia and sleep fragmentation may represent a potentially beneficial adjunct to cognitive rehabilitation in PwMS.

## Figures and Tables

**Table 1:** Summary of included studies.

Study	n	Inclusion Criteria	Exclusion Criteria	OSA	Cognitive Tests	Cognitive Domains	Results
Sater et al. 2015 [24]	32	EDSS < 6 ESS > 9 MFIS > 30 FSS > 4	BDI-II ≥ 32 Severe cognitive impairment Coexisting medical condition	37.5%	NeuroTrax	Memory Executive function Verbal fluency Attention Information processing speed	SE was correlated with executive function, information processing and global score. SOL was negatively associated with attention and executive function. WASO was correlated with verbal memory. There was no correlation between sleep apnea parameters and cognition.
Braley et al. 2016 [5]	38	Patients with sleep complaint	Severe cognitive impairment Severe immobility PHQ-9 ≥ 19 Visual or hearing impairments Treatment for OSA Drug or alcohol dependence Cardiopulmonary disease Other neurological disorder increasing OSA risk (stroke, Parkinson’s disease)	29%	MACFIMS (SDMT, PASAT, CVLT II, BVMT-R, JLO, COWAT, D-KEFS)	Information processing speed Working memory Executive function Visuospatial processing Language function	ODI, MinO_2_ were correlated with attention, information processing speed and visual memory. RDI was associated with attention, information processing speed and verbal memory. TAI and TST were associated with verbal memory. TST was also correlated with attention and information processing speed.
Chinnadurai et al. 2018 [25]	113	18–50 years	Psychiatric disease, dementia, alcohol or drug abuse, frequent night shift work BDI ≥ 32 EDSS > 6	15.04%	mStroop mSDMT SAT P300L P300A	Cognitive fatigue	SE, SOL, WASO, %N1, %N2, %N3, %REM were correlated with cognitive fatigue scores. SOL, %N1, %N2 were associated with electrophysiological cognitive fatigue.
McNicholas et al. 2021 [26]	23	Patients with important fatigue, impacting daily activities 18–60 years EDSS < 6.5	Relapse < 6 weeks Severe cognitive impairment Concomitant neurological disorder associated with OSA Treatment with CPAP or fatigue medication	30%	BICAMS (SDMT, CVLT-II, BVMT-R), PASAT-3	Information processing speed Verbal memory Visual memory Working memory	MinO_2_ was correlated with verbal memory. There was no correlation between sleep apnea parameters and cognition.
Riccitelli et al. 2022 [27]	80	Relapse- and steroid free for at least 1 month EDSS < 7 No change in DMT for at least 6 months	MMSE < 24 Diseases that cause cognitive impairment or PSG abnormalities History of drug or alcohol abuse Other neurological disease	-	BRB-N (SDMT, PASAT, SAT, SPART, WLG)	Information processing speed Attention Verbal memory Spatial memory Semantic fluency	SE and WASO were associated with attention, memory and global cognitive scores. %REM was positively associated with attention. There was no correlation between sleep apnea parameters and cognition.
Valentine et al. 2023 [6]	131	18–72 years OSA diagnosis or STOP-Bang ≥ 2	Physical, psychiatric, or cognitive impairment Cardiopulmonary conditions associated with OSA risk; Treatment for OSA Other nervous system diseased that could increase OSA risk or influence cognition; Pregnancy; Relapse or systemic high dose steroid use in the last 30 days; Anticipated medication changes; Any other condition or treatment that could affect participant safety or study eligibility	86.3%	MACFIMS (SDMT, PASAT, CVLT II, BVMT-R, JLO, COWAT) Trail Making Test Go/No-Go	Information processing speed Working memory Visual learning and memory Executive function Visuospatial processing Language function	%N2, %N3, SOL were correlated with verbal memory. %N2 and OAI were correlated with visual memory. ODI, OAI, CAI were associated with attention and information processing speed.
Maillart et al. 2024 [28]	71	EDSS ≥ 6.5 No relapse in the last 3 months	Severe COPD Pregnancy	27%	BICAMS (SDMT, CVLT, BVMT), MOCA	Information processing speed Verbal memory Visual memory Working memory	Sleep apnea was not correlated with cognitive performance.

Abbreviations: BDI—Beck Depression Inventory; BICAMS—Brief International Cognitive Assessment for Multiple Sclerosis; BRB-N—Brief Repeatable Battery of Neuropsychological Tests; BVMT—Brief Visuospatial Memory Test; CAI—Central Apnea Index; COPD—Chronic Obstructive Pulmonary Disease; COWAT—Controlled Oral Word Association Test; CPAP—Continuous Positive Airway Pressure; CVLT—California Verbal Learning Test; D-KEFS—Free Sort Test from the Delis-Kaplan Executive Function System; DMT—Disease-Modifying Therapy; EDSS—Expanded Disability Status Scale; ESS—Epworth Sleepiness Scale; FSS—Fatigue Severity Scale; JLO—Judgment of Line Orientation Test; MACFIMS—Minimal Assessment of Cognitive Function in Multiple Sclerosis; MFIS—Modified Fatigue Impact Scale; MinO_2_—Minimum Oxygen Saturation; MMSE—Mini Mental State Examination; MoCA—Montreal Cognitive Assessment; mSDMT—Modified Symbol Digit Modalities Test; mStroop—Modified Stroop Test; OAI—Obstructive Apnea Index; ODI—Oxygen Desaturation Index; OSA—Obstructive Sleep Apnea; P300A—Peak P300 Amplitude; P300L—Peak P300 Latency; PASAT—Paced Auditory Serial Addition Test; PHQ-9—Patient Health Questionnaire-9; PSG—Polysomnography; RDI—Respiratory Disturbance Index; REM—Rapid Eye Movement; SAT—Serial Addition Test; SDMT—Symbol Digit Modalities Test; SE—Sleep Efficiency; SOL—Sleep Onset Latency; SPART—10/36 Spatial Recall Test; TAI—Total Arousal Index; TST—Total Sleep Time; WASO—Wake After Sleep Onset; WLG—Word List Generation.

## Data Availability

No new data were created or analyzed in this study.

## Supplementary Materials

The following supporting information can be downloaded at: https://www.mdpi.com/article/10.3390/life16040699/s1, Table S1: Prisma 2020 checklist; Supplementary File S1: Complete search strategies for each database; Table S2: Studies excluded at full-text screening with reasons for exclusion; Table S3: NOS-xs quality assessment of included studies.

## Abbreviations

The following abbreviations are used in this manuscript:

AASM	American Academy of Sleep Medicine
AHI	Apnea–hypopnea index
BDI	Beck Depression Inventory
BDNF	Brain-derived neurotrophic factor
BRB-N	Brief Repeatable Battery of Neuropsychological Tests
BVMT	Brief Visuospatial Memory Test
BVMT-R	Brief Visuospatial Memory Test-Revised
BMI	Body Mass Index
COPD	Chronic obstructive pulmonary disease
COWAT	Controlled Oral Word Association Test
CPAP	Continuous positive airway pressure
CVLT	California Verbal Learning Test
CVLT-II	California Verbal Learning Test-II
D-KEFS	Delis-Kaplan Executive Function System
DMT	Disease-modifying therapy
EDSS	Expanded Disability Status Scale
ESS	Epworth Sleepiness Scale
FSS	Fatigue Severity Scale
HI	Hypopnea index
HPA	Hypothalamic–pituitary axis
IL-6	Interleukin-6
JLO	Judgment of Line Orientation
MFIS	Modified Fatigue Impact Scale
MinO_2_	Lowest oxygen saturation
MMSE	Mini Mental State Examination
MRI	Magnetic resonance imaging
MS	Multiple sclerosis
N1	Sleep stage N1
N2	Sleep stage N2
N3	Sleep stage N3
NOS-xs	Newcastle-Ottawa Scale for cross-sectional studies
NREM	Non-rapid eye movement
ODI	Oxygen desaturation index
OSA	Obstructive sleep apnea
PASAT	Paced Auditory Serial Addition Test
PHQ-9	Patient Health Questionnaire-9
PLMAI	Periodic limb movement arousal index
PLMI	Periodic limb movement index
PSG	Polysomnography
PwMS	People with multiple sclerosis
RDI	Respiratory disturbance index
REI	Respiratory event index
REM	Rapid eye movement
SAT	Serial Addition Test
SDMT	Symbol Digit Modalities Test
SE	Sleep efficiency
SOL	Sleep onset latency
SPART	Spatial Recall Test
SRT	Selective Reminding Test
TAI	Total arousal index
TNF-α	Tumor necrosis factor-alpha
TST	Total sleep time
WASO	Wake after sleep onset
WLG	Word list generation
