# Supplementary material for: Sleep Disorders and Cognitive Function in Multiple Sclerosis: A Systematic Review of Polysomnographic Studies and Implications for Neurorehabilitation Strategies
Source: Life (Basel). 2026 Apr 21;16(4):699. doi: 10.3390/life16040699 (PMC13117792; doi:10.3390/life16040699)
Supplement: Supplementary file 1 [file life-16-00699-s001.zip › File S1_Search_strategy.pdf]

Pubmed
#1 ("multiple sclerosis"[Title/Abstract] OR "MS" [Title/Abstract])
#2 ("polysomnography"[MeSH Terms] OR "polysomnography"[Title/Abstract] OR "PSG"[Title/Abstract] OR "sleep study"[Title/Abstract] OR "sleep apnea"[Title/Abstract] OR “sleep apnoea”[Title/Abstract] OR "sleep disorder*"[Title/Abstract] OR “sleep disturbance*”[Title/Abstract] OR "sleep impairment*"[Title/Abstract])
#3 (“cognitive dysfunction"[MeSH Terms] OR "cognitive dysfunction"[Title/Abstract] OR "cognitive impairment"[Title/Abstract] OR cogni*[Title/Abstract] OR "Cognition"[Mesh])
#4 #1 AND #2 AND #3

EMBASE
#1 'multiple sclerosis':ti,ab OR 'MS':ti,ab
#2 'polysomnography':ti,ab OR 'psg':ti,ab OR 'sleep study':ti,ab OR 'sleep apnea':ti,ab OR 'sleep apnoea':ti,ab OR 'sleep disorder*':ti,ab OR 'sleep disturbance*':ti,ab OR 'sleep impairment*':ti,ab
#3 'cognitive dysfunction':ti,ab OR 'cognitive impairment':ti,ab OR cogni*:ti,ab
#1 AND #2 AND #3

Cochrane Library
#1 MeSH descriptor: [Multiple Sclerosis] this term only
#2 ("multiple sclerosis"):ti,ab,kw OR (MS):ti,ab,kw
#3 MeSH descriptor: [Polysomnography] explode all trees
#4 (polysomnography):ti,ab,kw OR (PSG):ti,ab,kw OR (sleep NEXT (study OR apnea OR apnoea OR disorder* OR disturbance* OR impairment*)):ti,ab,kw
#5 MeSH descriptor: [Cognition] explode all trees
#6 MeSH descriptor: [Cognitive Dysfunction] explode all trees
#7 (cogniti*):ti,ab,kw OR (cognitive NEXT (dysfunction OR impairment)):ti,ab,kw
#8 #1 OR #2
#9 #3 OR #4
#10 #5 OR #6 OR #7
#11 #8 AND #9 AND #10
