# Supplementary material for: Sleep Disorders and Cognitive Function in Multiple Sclerosis: A Systematic Review of Polysomnographic Studies and Implications for Neurorehabilitation Strategies
Source: Life (Basel). 2026 Apr 21;16(4):699. doi: 10.3390/life16040699 (PMC13117792; doi:10.3390/life16040699)
Supplement: Supplementary file 1 [file life-16-00699-s001.zip › Table S2_Full-text excluded studies.pdf]

Article	Primary reason for exclusion
Petrescu et al. 2025 [1]	Subjective evaluation of sleep
Kaya et al. 2025 [2]	Subjective evaluation of sleep
Wang et al. 2025 [3]	Subjective evaluation of sleep
Khedr et al. 2025 [4]	Subjective evaluation of sleep
Ashtari et al. 2025 [5]	Subjective evaluation of sleep
Aparicio-Castro et al. 2025 [6]	Subjective evaluation of sleep
Rzepka et al. 2024 [7]	Sleep-cognition relationship not reported
Vural et al. 2024 [8]	Sleep-cognition relationship not reported
Paniagua Gonzalez et al. 2024 [9]	Actigraphy
Hersh et al. 2024 [10]	Subjective evaluation of sleep and cognition
Terauchi et al. 2024 [11]	Actigraphy
Mazerolle et al. 2024 [12]	Sleep-cognition relationship not reported
Sempik et al. 2024 [13]	Subjective evaluation of sleep
Ozdogar et al. 2024 [14]	Subjective evaluation of sleep
Ottersbach et al. 2023 [15]	Sleep-cognition relationship not reported
Braley et al. 2023 [16]	Subjective evaluation of sleep and cognition
Cederberg et al. 2022 [17]	Subjective evaluation of sleep
Whibley et al. 2021 [18]	Actigraphy
Al-Sharman et al. 2021 [19]	Actigraphy
Sumowski et al. 2021 [20]	Subjective evaluation of sleep
Dubessy et al. 2021 [21]	Sleep-cognition relationship not reported
Odintsova et al. 2021 [22]	Subjective evaluation of sleep
Cederberg et al. 2020 [23]	Subjective evaluation of sleep and cognition
Türkoğlu et al. 2020 [24]	Sleep-cognition relationship not reported
Vega et al. 2019 [25]	Subjective evaluation of sleep
Borragán et al. 2018 [26]	Sleep-cognition relationship not reported
Sadeghi Bahmani et al. 2018 [27]	Subjective evaluation of sleep
Aldughmi et al. 2016 [28]	Actigraphy
Siengsukon et al. 2018 [29]	Subjective evaluation of sleep
Patel et al. 2017 [30]	Subjective evaluation of sleep
Hughes et al. 2017 [31]	Subjective evaluation of sleep
van Geest et al. 2017 [32]	Subjective evaluation of sleep
Al-Dughmi et al. 2016 [33]	Actigraphy
Sater et al. 2016 [34]	Sleep-cognition relationship not reported

Table S2. Full text excluded studies with reasons for exclusion.

References

1. Petrescu, S.; Dumitru-Martoiu, M.M.; Panea, C.A. Multidimensional Impairment in Multiple Sclerosis: Physical Disability, Cognitive Dysfunction, Sleep Disturbance, Fatigue, Depression, and Their Impact on Quality of Life-A Possible Common Pathological Pathway. *Neurol. Int.* 2025, 17, 174. https://doi.org/10.3390/neurolint17110174
2. Kaya, A.; Karadağ-Saygı, E.; Kucukosmanoglu, Z.; Keniş-Coşkun, Ö.; Ağan Yıldırım, K. Effect of Telerehabilitation Exercise Program on Sleep Quality and Fatigue in Individuals With Multiple Sclerosis. *Ann. Rehabil. Med.* 2025, 49, 302–309. https://doi.org/10.5535/arm.250010

3. Wang, R.; Zhang, T.; Wang, H.; Ren, Y.; Zhao, R.; Zhang, G.; Zhang, G.; Zhao, X. Characteristics and correlations of sleep disorders in patients with relapsing-remitting multiple sclerosis in China: a cross-sectional study. *Front. Neurol.* 2025, 16, 1608802. Erratum in: *Front. Neurol.* 2025, 16, 1669440. https://doi.org/10.3389/fneur.2025.1608802
4. Khedr, E.M.; Ahmed, G.K.; Hassan, S.S.; Foly, M.N.; Attia, M.M.; Karim, A.A.; Haridy, N.A. Frequency and risk factors of sleep problems in Egyptian patients with multiple sclerosis. *Front. Neurol.* 2025, 16, 1563041. https://doi.org/10.3389/fneur.2025.1563041
5. Ashtari, F.; Ghalamkari, A.; Naghavi, S.; Pourmohammadi, A.; Adibi, I.; Karimi, Z.; Kavosh, A. Relationship between sleep disorders and information processing speed in multiple sclerosis. *Clinics (Sao Paulo)* 2025, 80, 100574. https://doi.org/10.1016/j.clinsp.2024.100574
6. Aparicio-Castro, E.; Candeliere-Merlicco, A.; Santa, C.M.; Villaverde-González, R. Association of depression in multiple sclerosis with fatigue, sleep disturbances, disability, and health-related quality of life: Outcomes of a cross-sectional study. Neurol. Perspect. 2025, 5, 100181. https://doi.org/10.1016/j.neurop.2024.100181
7. Rzepka, M.; Chmiela, T.; Galus, W.; Lasek-Bal, A.; Krzystanek, E. Exploring Sleep Architecture in Polish Patients with Multiple Sclerosis: A Polysomnography Study. *Brain Sci.* 2024, 14, 932. https://doi.org/10.3390/brainsci14090932
8. Vural, G.; Demir, E.; Gumusyayla, S.; Eren, F.; Barakli, S.; Neselioglu, S.; Erel, O. The Role of the Lipid Profile and Oxidative Stress in Fatigue, Sleep Disorders and Cognitive Impairment in Patients with Multiple Sclerosis. *Acta Med. Okayama* 2024, 78, 259–270. https://doi.org/10.18926/AMO/67201
9. Paniagua Gonzalez, L.; Eichau, S.; Ortega Carrión, L.; Borges, M.; Domínguez, E.; López Ruiz, R. ACTIVE-FIT program: Assessment of sleep quality and its relationship with physical activity in patients with relapsing-remitting multiple sclerosis. *Sleep Med.* 2024, 119, 373–378. https://doi.org/10.1016/j.sleep.2024.05.005
10. Hersh, C.M.; Pang, M.; Miller, D.M.; McGinley, M.P.; Hyland, M.; Ziemssen, T.; Avila, R.L. Comparison of time to clinically meaningful improvement in quality of life in neurological disorders in patients treated with natalizumab versus ocrelizumab. *Neurodegener. Dis. Manag.* 2024, 14, 21–33. https://doi.org/10.2217/nmt-2023-0047
11. Terauchi, T.; Mizuno, M.; Suzuki, M.; Akasaka, H.; Maeta, M.; Tamura, K.; Hosokawa, K.; Nishijima, T.; Maeda, T. Clinical features of sleep apnea syndrome and cognitive impairment in multiple sclerosis. *Mult. Scler. Relat. Disord.* 2024, 82, 105407. https://doi.org/10.1016/j.msard.2023.105407
12. Mazerolle, M.; Kimoff, R.J.; Khadadah, S.; Duquette, P.; Jobin, V.; Lapierre, Y.; Benedetti, A.; Majetic, K.; Robinson, A.; Roger, E.; Bar-Or, A.; Kaminska, M.; Leonard, G.; Trojan, D.A. Long term effects of continuous positive airway pressure treatment of obstructive sleep apnea-hypopnea syndrome in multiple sclerosis patients. *Mult. Scler. Relat. Disord.* 2024, 81, 105144. https://doi.org/10.1016/j.msard.2023.105144
13. Sempik, I.; Dziadkowiak, E.; Wieczorek, M.; Pokryszko-Dragan, A. Sleep disturbance and related factors in the patients with relapsing-remitting multiple sclerosis. Acta Neurol. Scand. 2024, 2024, 6656571. https://doi.org/10.1155/2024/6656571
14. Ozdogar, A.T.; Aldemir, E.; Yesiloglu, P. Exploring the relationship between sleep quality and fatigue, quality of life, daytime sleepiness, and anxiety-depression levels in patients with multiple sclerosis. J. Mult. Scler. Res. 2024, 4, 67–72. https://doi.org/10.4274/jmsr.galenos.2024.2024-12-2

15. Ottersbach, J.; Wetter, T.C.; König, N.; Fierlbeck, A.; Weissert, R.; Popp, R.F. Prospective analyses of alertness, sleep, and fitness to drive one year after de novo multiple sclerosis diagnosis. *Mult. Scler. Relat. Disord.* 2023, 79, 104930. https://doi.org/10.1016/j.msard.2023.104930
16. Braley, T.J.; Shieu, M.M.; Zaheed, A.B.; Dunietz, G.L. Pathways between multiple sclerosis, sleep disorders, and cognitive function: Longitudinal findings from The Nurses' Health Study. *Mult. Scler.* 2023, 29, 436–446. https://doi.org/10.1177/13524585221144215
17. Cederberg, K.L.J.; Mathison, B.; Schuetz, M.L.; Motl, R.W. Restless Legs Syndrome Severity and Cognitive Function in Adults With Multiple Sclerosis: An Exploratory Pilot Study. *Int. J. MS Care* 2022, 24, 154–161. https://doi.org/10.7224/1537-2073.2020-120
18. Whibley, D.; Goldstein, C.; Kratz, A.L.; Braley, T.J. A multidimensional approach to sleep health in multiple sclerosis. *Mult. Scler. Relat. Disord.* 2021, 56, 103271. https://doi.org/10.1016/j.msard.2021.103271
19. Al-Sharman, A.; Al-Khazaaleh, H.M.; Khalil, H.; Aburub, A.; El-Salem, K. The Relationship Between Sleep Quality, Sleep-Related Biomarkers, and Motor Skill Acquisition in People With Multiple Sclerosis: A Pilot Study. *Phys. Ther.* 2021, 101, pzab175. https://doi.org/10.1093/ptj/pzab175
20. Sumowski, J.F.; Horng, S.; Brandstadter, R.; Krieger, S.; Leavitt, V.M.; Katz Sand, I.; Fabian, M.; Klineova, S.; Graney, R.; Riley, C.S.; Lublin, F.D.; Miller, A.E.; Varga, A.W. Sleep disturbance and memory dysfunction in early multiple sclerosis. *Ann. Clin. Transl. Neurol.* 2021, 8, 1172–1182. https://doi.org/10.1002/acn3.51262
21. Dubessy, A.L.; Tezenas du Montcel, S.; Viala, F.; Assouad, R.; Tiberge, M.; Papeix, C.; Lubetzki, C.; Clanet, M.; Arnulf, I.; Stankoff, B. Association of Central Hypersomnia and Fatigue in Patients With Multiple Sclerosis: A Polysomnographic Study. *Neurology* 2021, 97, e23–e33. https://doi.org/10.1212/WNL.0000000000012120
22. Odintsova, T.A.; Kopchak, O.O. Sleep Disorders in Relapsing-Remitting Multiple Sclerosis Patients. *Wiad. Lek.* 2021, 74, 257–262. https://doi.org/10.36740/WLek202102115
23. Cederberg, K.L.J.; Jeng, B.; Sasaki, J.E.; Motl, R.W. Restless legs syndrome, sleep quality, and perceived cognitive impairment in adults with multiple sclerosis. *Mult. Scler. Relat. Disord.* 2020, 43, 102176. https://doi.org/10.1016/j.msard.2020.102176
24. Türkoğlu, R.; Benbir, G.; Özyurt, S.; Arsoy, E.; Akbayır, E.; Turan, S.; Karadeniz, D.; Yılmaz, V.; Gencer, M.; Tüzün, E. Sleep disturbance and cognitive decline in multiple sclerosis patients with isolated optic neuritis as the first demyelinating event. *Int. Ophthalmol.* 2020, 40, 151–158. https://doi.org/10.1007/s10792-019-01157-x
25. Vega, R.; Miró, J.; Esteve, R.; Ramírez-Maestre, C.; López-Martínez, A.E.; Jensen, M.P. Sleep disturbance in individuals with physical disabilities and chronic pain: The role of physical, emotional and cognitive factors. Disabil. Health J. 2019, 12, 588–593. https://doi.org/10.1016/j.dhjo.2019.04.001
26. Borragán, G.; Gilson, M.; Atas, A.; Slama, H.; Lysandropoulos, A.; De Schepper, M.; Peigneux, P. Cognitive Fatigue, Sleep and Cortical Activity in Multiple Sclerosis Disease. A Behavioral, Polysomnographic and Functional Near-Infrared Spectroscopy Investigation. Front. Hum. Neurosci. 2018, 12, 378. https://doi.org/10.3389/fnhum.2018.00378
27. Sadeghi Bahmani, D.; Esmaeili, L.; Shaygannejad, V.; Gerber, M.; Kesselring, J.; Lang, U.E.; Holsboer-Trachsler, E.; Brand, S. Stability of Mental Toughness, Sleep Disturbances, and Physical Activity in Patients With Multiple Sclerosis (MS)-A

Longitudinal and Pilot Study. Front. Psychiatry 2018, 9, 182. https://doi.org/10.3389/fpsyt.2018.00182
28. Aldughmi, M.; Huisinga, J.; Lynch, S.G.; Siengsukon, C.F. The relationship between fatigability and sleep quality in people with multiple sclerosis. Mult. Scler. J. Exp. Transl. Clin. 2016, 2, 2055217316682774. https://doi.org/10.1177/2055217316682774
29. Siengsukon, C.F.; Aldughmi, M.; Kahya, M.; Lynch, S.; Bruce, J.; Glusman, M.; Ness Norouzinia, A.; Billinger, S. Individuals with mild MS with poor sleep quality have impaired visuospatial memory and lower perceived functional abilities. Disabil. Health J. 2018, 11, 116–121. https://doi.org/10.1016/j.dhjo.2017.04.011
30. Patel, V.P.; Walker, L.A.; Feinstein, A. Processing speed and distractibility in multiple sclerosis: the role of sleep. *Mult. Scler. Relat. Disord.* 2017, 11, 40–42. https://doi.org/10.1016/j.msard.2016.11.012
31. Hughes, A.J.; Parmenter, B.A.; Haselkorn, J.K.; Lovera, J.F.; Bourdette, D.; Boudreau, E.; Cameron, M.H.; Turner, A.P. Sleep and its associations with perceived and objective cognitive impairment in individuals with multiple sclerosis. *J. Sleep Res.* 2017, 26, 428–435. https://doi.org/10.1111/jsr.12490
32. van Geest, Q.; Westerik, B.; van der Werf, Y.D.; Geurts, J.J.; Hulst, H.E. The role of sleep on cognition and functional connectivity in patients with multiple sclerosis. *J. Neurol.* 2017, 264, 72–80. https://doi.org/10.1007/s00415-016-8318-6
33. Al-Dughmi, M.; Siengsukon, C.F. The relationship between sleep quality and perceived fatigue measured using the Neurological Fatigue Index in people with Multiple Sclerosis. *Neurol. Res.* 2016, 38, 943–949. https://doi.org/10.1080/01616412.2016.1232014
34. Sater, R.A.; Gudesblatt, M.; Kresa-Reahl, K.; Brandes, D.W.; Sater, P. NAPS-MS: Natalizumab Effects on Parameters of Sleep in Patients with Multiple Sclerosis. *Int. J. MS Care* 2016, 18, 177–182. https://doi.org/10.7224/1537-2073.2015-033
