# Supplementary material for: Sleep Disorders and Cognitive Function in Multiple Sclerosis: A Systematic Review of Polysomnographic Studies and Implications for Neurorehabilitation Strategies
Source: Life (Basel). 2026 Apr 21;16(4):699. doi: 10.3390/life16040699 (PMC13117792; doi:10.3390/life16040699)
Supplement: Supplementary file 1 [file life-16-00699-s001.zip › Table S3_NOS-xs.pdf]

Study	Study Sample Selection (out of 2)	Confounding Factors (out of 3)	Outcome (out of 4)	NOS-xs total (out of 9)
Sater et al. 2015 [24]	☆	☆☆	☆☆☆	☆☆☆☆☆☆
Braley et al. 2016 [5]	☆	☆☆☆	☆☆☆☆	☆☆☆☆☆☆☆☆
Chinnadurai et al. 2018 [25]	☆☆		☆☆☆	☆☆☆☆☆
McNicholas et al. 2021 [26]	☆	☆☆	☆☆☆☆	☆☆☆☆☆☆☆
Riccitelli et al. 2022 [27]	☆☆	☆☆☆	☆☆☆	☆☆☆☆☆☆☆☆
Valentine et al. 2023 [6]	☆	☆☆☆	☆☆☆☆	☆☆☆☆☆☆☆☆
Maillart et al. 2024 [28]	☆		☆☆☆☆	☆☆☆☆☆

Table S3. Newcastle-Ottawa Scale for cross-sectional studies (NOS-xs) quality assessment of included studies. Full scoring criteria: Carra, M.C.; Romandini, P.; Romandini, M. Risk of bias evaluation of cross-sectional studies: Adaptation of the Newcastle-Ottawa Scale. J. Periodontal Res. 2025, ahead of print. https://doi.org/10.1111/jre.13405.
